# Clustered regularly interspaced short palindromic repeats/CRISPR-associated protein 9-generated diallelic mutants reveal Arabidopsis actin-related protein 2 function in the trafficking of syntaxin PEN1

**DOI:** 10.3389/fpls.2022.934002

**Published:** 2022-09-20

**Authors:** Peng Gao, Li Qin, Hanh Nguyen, Huajin Sheng, Teagen D. Quilichini, Daoquan Xiang, Leon V. Kochian, Yangdou Wei, Raju Datla

**Affiliations:** ^1^Global Institute for Food Security, University of Saskatchewan, Saskatoon, SK, Canada; ^2^Agriculture and Agri-Food Canada, Saskatoon Research and Development Centre, Saskatoon, SK, Canada; ^3^Aquatic and Crop Resource Development, National Research Council Canada, Saskatoon, SK, Canada; ^4^College of Arts and Science, University of Saskatchewan, Saskatoon, SK, Canada

**Keywords:** CRISPR/Cas9, actin-related protein, diallelic mutants, somatic mutations, plant pathogen interactions

## Abstract

In plants, the actin cytoskeleton plays a critical role in defense against diverse pathogens. The formation of actin patches is essential for the intracellular transport of organelles and molecules toward pathogen penetration sites and the formation of papillae for an early cellular response to powdery mildew attack in *Arabidopsis thaliana*. This response process is regulated by the actin-related protein (ARP)2/3 complex and its activator, the WAVE/SCAR complex (W/SRC). The ARP2/3 complex is also required for maintaining steady-state levels of the defense-associated protein, PENETRATION 1 (PEN1), at the plasma membrane and for its deposition into papillae. However, specific ARP2 functionalities in this context remain unresolved, as knockout mutants expressing *GFP-PEN1* reporter constructs could not be obtained by conventional crossing approaches. In this study, employing a CRISPR/Cas9 multiplexing-mediated genome editing approach, we produced an ARP2 knockout expressing the *GFP-PEN1* marker in *Arabidopsis*. This study successfully identified diallelic somatic mutations with both *ARP2* alleles edited among the primary T1 transgenic plants, and also obtained independent lines with stable *arp2/arp2* mutations in the T2 generation. Further analyses on these *arp2/arp2* mutants showed similar biological functions of *ARP2* to *ARP3* in the accumulation of PEN1 against fungal invasion. Together, this CRISPR/Cas9-based approach offers highly efficient simultaneous disruption of the two *ARP2* alleles in *GFP-PEN1*-expressing lines, and a rapid method for performing live-cell imaging to facilitate the investigation of important plant–pathogen interactions using a well-established and widely applied GFP marker system, thus gaining insights and elucidating the contributions of ARP2 upon fungal attack.

## Introduction

Sessile plants have evolved multilayered defense mechanisms to protect against the attack and colonization of pathogens, such as bacteria, fungi, and oomycetes. To defend against biotrophic powdery mildew fungi, plants employ a defense response involving at least two tiers, known as penetration resistance and post-invasion resistance, the latter of which is typically associated with the hypersensitive response of invaded host cells (Thordal-Christensen, 2003; Lipka et al., 2005). Penetration resistance is achieved by the development of a thickened waxy cuticle and cell wall, and by the inducible formation of cell wall appositions (known as papillae) in the paramural space subtending the cell wall, which is thought to provide physical barriers beneath attempted entry sites that halt fungal penetration peg invasions (Hückelhoven, 2007; Kuhn et al., 2016). Apoplastic papillae contain callose, lignin, reactive oxygen species, and proteins PENETRATION (PEN) 1-4 (Thordal-Christensen et al., 1997; Assaad et al., 2004; Lipka et al., 2005; Stein et al., 2006; Meyer et al., 2009; Hématy et al., 2020). Syntaxin PEN1/SYP121 is required for the localized formation of ternary SNARE complexes and fusion of vesicles with the plasma membrane (Assaad et al., 2004; Kwon et al., 2008). Myrosinase PEN2 and phytochelatin synthase PEN4/PCS1 generate toxic hydrolysis products of indole glucosinolates (Lipka et al., 2005; Bednarek et al., 2009; Clay et al., 2009; Hématy et al., 2020). ABC transporter PEN3 mobilizes these toxic secondary metabolites across the plasma membrane to the apoplastic region (Stein et al., 2006). All PEN proteins show focal accumulation at penetration sites upon powdery mildew attack, although PEN1 and PEN3 primarily localize at the plasma membrane while PEN2 and PEN4 are targeted to mitochondria (Assaad et al., 2004; Underwood and Somerville, 2013).

The actin cytoskeleton plays a pivotal role in various plant–microbe interactions, mainly by providing tracks for long-distance and polar transport processes (Li and Staiger, 2018). Disruption of the actin cytoskeleton by pharmacological or genetic interference impairs host and non-host resistance of plants against a broad range of fungal and oomycete pathogens (Takemoto et al., 2003, 2006; Yun et al., 2003; Yang et al., 2014). The focal deposition of papillary materials and the accumulation of intracellular compartments at attempted penetration sites are thought to be orchestrated by the polar actin network (Hardham et al., 2007; Underwood and Somerville, 2008). In plant–powdery mildew interactions, attempted penetration of the cell wall triggers sequential responses of rapid actin rearrangement, which is characterized by two disparate actin architectures: actin patches and actin bundles. Initially, the host cell forms an actin patch focally surrounding the penetration site, and later radial actin bundles orient transversely across the infected cell with enrichment at the penetration site anchored at one end to the actin patch (Yang et al., 2014; Qin et al., 2021).

In all eukaryotic cells, actin networks undergo rapid remodeling, which is assisted by a range of actin-binding proteins (Moseley and Goode, 2006; Pollard, 2016). Among these actin-binding proteins, the actin-related protein (ARP)2/3 complex and formin proteins are known to directly participate in the nucleation of new filaments (Pollard, 2007). Conserved among eukaryotes, the ARP2/3 complex consists of seven polypeptides, including ARP2, ARP3, and five additional subunits (named ARPC1 to ARPC5). In the presence of ATP and nucleation-promoting factors, the ARP2/3 complex binds to the side of pre-existing actin filaments and initiates the filament branch, in which ARP2 and ARP3 are proposed to form the first two subunits of the daughter filament (Rouiller et al., 2008).

Although ARP2 and ARP3 are the key subunits of the ARP2/3 complex and are indispensable in the initiation of branched filaments, they exhibit some notable differences. For example, *in vivo* nucleotide-binding test showed that actin patches in yeast *arp3*-G302Y mutant become almost completely depolarized when shifted to the restrictive temperature of 37°C, while actin patches in *arp2*-G302Y mutant are polarized at both 25 and 37°C. Similarly, the *arp3*-G302Y mutants are significantly impaired in Lucifer yellow uptake (an indicator of endocytic function), while *arp2*-G302Y only exhibits moderate reduction, which indicates the importance of ARP3 over ARP2 for endocytosis in yeast. In addition, only ARP3 is necessary to assemble the ARP2/3 complex, since fission yeast that lacks ARP2 can still form the complex and has all of the other subunits in their normal positions (Nolen and Pollard, 2008). The actin patches formed in Arabidopsis–powdery mildew interactions are assembled by the ARP2/3 complex, with the help of Class I formins (Qin et al., 2021). Our previous study showed that maintaining the steady-state level of PEN1 at the plasma membrane and accumulation of GFP-PEN1 at papillae are partially impaired in null *arp3* mutants (Qin et al., 2021). However, this study did not address the direct role of ARP2 in the trafficking of PEN1. Attempts to generate homozygous *arp2* plants expressing GFP-PEN1 in the F2 generation were not successful after screening thousands of seedlings from the cross of GFP-PEN1 and null *arp2* T-DNA mutant, probably for multiple reasons, for example, lethality or linkage between the gene *ARP2* and the inserted *GFP-PEN1* fragment.

Clustered regularly interspaced short palindromic repeats/CRISPR-associated protein 9 (CRISPR/Cas9) is a rapidly emerging powerful approach for genome engineering that seeks to efficiently modify target DNA in diverse species including plants (Jinek et al., 2012; Wiedenheft et al., 2012; Fauser et al., 2014). The CRISPR/Cas9 genome editing system contains two key functional components: the Cas9 endonuclease and the guide RNA (gRNA), which is complementary and binds to a specific target DNA sequence that ends with a short DNA sequence, known as the protospacer adjacent motif (PAM) (Ran et al., 2013). When used for genome editing, CRISPR/Cas9 nuclease-based gene drives can create a targeted DNA double-strand break (DSB) in a genome. DNA repair then achieves the desired DNA sequence modification by non-homologous end joining (NHEJ), which can be used to create insertion/deletion (indel) mutations, gene replacements, and single base pair conversions at the break site (Feng et al., 2013; Xie and Yang, 2013; Pandey et al., 2019). CRISPR/Cas9-mediated gene knockouts have been successfully produced in diverse plant species that are amenable to gene delivery and transformation to manipulate genetic pathways to improve desirable agronomic traits, and develop pathogen-resistant crops (Brooks et al., 2014; Jiang et al., 2017; Zong et al., 2017; Pandey et al., 2019). Moreover, exploitation of the CRISPR/Cas9 multiplexing capability has made it possible to edit multiple genes or target a single gene using multiple gRNAs simultaneously within one or two generations. This approach can also be applied to knock-out genes that are closely linked. During the process of CRISPR/Cas9 gene editing, the use of constitutive promoters can favor the generation of somatic mutations over mutations in the germlines of reproductive tissues, due to relatively lower expression in the meristems and early developmental stages of embryogenesis than in the mature tissues and organs (Feng et al., 2014). To enrich for inheritable mutations, germline-specific Arabidopsis promoters including the egg cell–specific promoters EC1.1, EC1.2, and the Yao promoters have been applied successfully (Wang et al., 2015; Yan et al., 2015). Although somatic mutations are less heritable than germline mutations, they can generate diverse mutant types in mature differentiated tissues, providing opportunities to investigate primary T1 generation of transgenics for CRISPR-mediated mutations in an array of cell types and tissues during vegetative stages of plant development.

In this study, we applied a multiplexing CRISPR gene-editing approach (Čermák et al., 2017) and generated multiple unique somatic diallelic heterozygous *arp2/arp2* mutations in GFP-PEN1-expressing lines. The T1 transgenic plants produced in this study show a range of trichome morphology defects and GFP-PEN1 signals at the plasma membrane and at powdery mildew attack-induced papillae, which were further confirmed and validated by analyzing T2 stable homozygous transgenic plants. The findings from this study provide evidence that ARP2, along with ARP3, plays an important role in maintaining the steady-state level of GFP-PEN1 at the plasma membrane and in the transport of GFP-PEN1 into papillae.

## Results

### A clustered regularly interspaced short palindromic repeats/CRISPR-associated protein 9-mediated *ARP2* gene editing in GFP-PEN1-expressing lines

Our previous work showed that the actin-related protein ARP3 and its activator, the WAVE/SCAR complex (W/SRC) are required for maintaining steady-state levels of the defense-associated protein, PENETRATION 1 (PEN1), at the plasma membrane (Qin et al., 2021). Knockout of the W/SRC–ARP2/3 pathway subunit, ARP3, partially compromised penetration resistance with impaired endocytic recycling of PEN1 and its deposition into apoplastic papillae. However, investigating the specific functionalities of ARP2, another W/SRC-ARP2/3 pathway subunit, in plants expressing *GFP-PEN1* could not be achieved by conventional crossing between the *arp2* SALK mutant line and GFP-PEN1-expressing lines. This is probably due to the close genetic linkage between *ARP2* and the GFP-PEN1 insertion or some other causes yet to be defined. Considering that this GFP-PEN1-expressing transgenic line is a well-established and widely used tool for the last 20 years, and generating new transgenic GFP-PEN1-expressing lines without genetic linkage to *ARP2* might cause additional complexities or provide inconsistent results, we, therefore, applied the powerful CRISPR/Cas9 genome editing approaches to develop direct *ARP2* gene editing, to generate targeted mutations at this locus in the elite GFP-PEN1-expressing line.

Toward this, we employed the classic CRISPR/Cas9 multiplexing-mediated approach and selected the backbone binary vector containing a Csy-type (CRISPR system yersinia) ribonuclease 4 (Csy4) spacer and the *Cestrum Yellow Leaf Curling Virus* (CmYLCV) promoter, which offers many advantages, like higher effective editing frequency, shorter spacer, and non-duplicated promoters (Čermák et al., 2017). We found that this construct could generate potential diallelic heterozygous mutations in the target genes. Toward this, three gRNAs were designed to target *ARP2*, for which the specificity to distinguish *ARP3* and other genes with a highly similar sequence was considered. All these components were assembled into the pDIRECT22C vector toolkit (Čermák et al., 2017) to create the final CRISPR vector for editing the *ARP2* gene in Arabidopsis.

**FIGURE 1:**
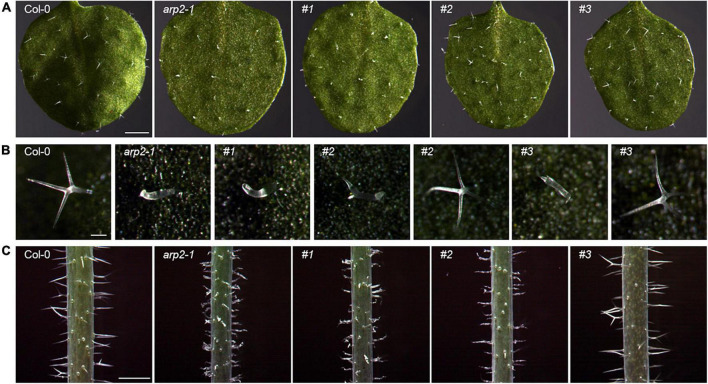
Trichome phenotypes of *Col-0*, *arp2* T-DNA mutant, and CRISPR transgenic lines with GFP-PEN1 expressing background. **(A)** Overview of trichomes on the first true leaf from 2-week-old plants. Bars, 1 mm. **(B)** Single trichomes on the first true leaf from 2-week-old plants. Bars, 100 μm. **(C)** Trichomes on stems from 4-week-old plants. Bars, 1 μm. #1, #2, and #3 indicate three individual T1 generation CRISPR transgenic lines in the GFP-PEN1 expressing background.

After Agrobacterium-mediated transformation of the CRISPR vector into Col-0 wild-type and GFP-PEN1-expressing plants, positive T1 seeds were obtained with kanamycin selection. All T1-positive transformants seedlings appeared phenotypically similar in overall morphology to the wild-type at 2 weeks old. However, some positive T1 transgenic plants under both Col-0 and GFP-PEN1-expressing backgrounds had significant trichome morphology defects on the first true leaves (Figure 1A), similar to previously described phenotypes for ARP2/3 complex and SCAR complex subunit mutants (Djakovic et al., 2006). Closer observation revealed variations in the degree of trichome branching, including trichomes where branching failed to initiate or initiated but failed to fully elongate (Figure 1B, plants #1, #2, and #3), with swollen stalks that occasionally appeared excessively elongated relative to wild-type. These trichome abnormalities phenotypically resembled *arp2-1* T-DNA SALK lines. Interestingly, two phenotypes in T1-positive transformants were identified, including completely aberrant trichomes (Figure 1B, plant #1) on the first true leaves, as in SALK mutant line *arp2-1*; and a mix of abnormal and normal trichomes on the first true leaves (Figure 1B, plants #2 and #3). Similar phenotypic diversity was also observed among the trichomes on 4-week-old stems, with completely abnormal trichomes on transgenic plant #1 and partially abnormal trichomes on transgenic plants #2 and #3 (Figure 1C). Since the abnormal trichome phenotype is recessive and was visible on T1 transgenic plants, it is possible that a diallelic mutation created by CRISPR editing of these plants, generating the loss of function of both alleles of the *ARP2* locus and the observed mutant phenotype.

### Actin-related protein 2 is required to maintain steady-state levels of PEN1 at the plasma membrane

**FIGURE 2:**
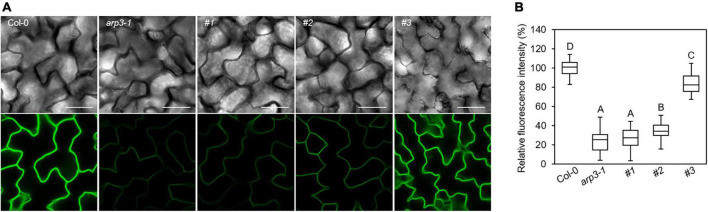
Clustered regularly interspaced short palindromic repeats (CRISPR)-derived gene variants of *ARP2* exhibit different levels of GFP-PEN1 in leaf epidermal cells. **(A)** Representative confocal images of GFP-PEN1 signals in the epidermal cells of the first true leaves of 2-week-old wild-type *Col-0*, *arp3-1* T-DNA mutant line, and T1 generation of CRISPR transgenic individual lines #1, #2, and #3 in GFP-PEN1 expressing plants. Micrographs were captured with the same imaging parameters. Bars, 100 μm. **(B)** Boxplots displaying relative fluorescence intensity of GFP-PEN1 at the plasma membrane in the indicated genotypes. Quantitative analysis was performed for 30 cells per genotype. Different letters indicate statistically significant differences, as determined by one-way ANOVA with Tukey’s HSD (*p* < 0.01).

The CRISPR/Cas9 generated loss-of-function *ARP2* transgenic plants in the GFP-PEN1-expressing background, and created the opportunity to investigate the roles and impact of ARP2 on the subcellular trafficking and accumulation of PEN1 in Arabidopsis. The signal of GFP-PEN1 at the plasma membrane in epidermal cells was significantly decreased in the *arp3-1* T-DNA mutant lines (Figure 2). Confocal imaging revealed dramatic reductions in GFP-PEN1 signals at the plasma membrane of leaf epidermal cells on the same first true leaves of CRISPR transgenic lines, to only 26% (plant #1) or 35% (plant #2) of wild-type levels (Figures 2A,B). This result supports a critical role for ARP2 in maintaining a steady-state level of GFP-PEN1 at the PM, as observed for ARP3. A moderate decrease of mean GFP-PEN1 signal intensity was observed in #3 plant (84% of the level at the wild-type, Figure 2B), and in some epidermal cells the GFP-PEN1 signal intensity maintained a similar level to that in the wild-type, suggesting that the function of ARP2 is not disrupted in these cells. The variation in the GFP-PEN1 signal detected in different CRISPR transgenic lines correlated with the severity of trichome phenotypic variation, with the weaker GFP-PEN1 intensity observed in completely abnormal trichome lines and the stronger GFP-PEN1 intensity observed in partially abnormal trichome lines. The relatively normal GFP-PEN1 signals and trichome development phenotypes observed in transgenic plant #3 suggest the existence of functional ARP2 in some epidermal cells, either due to the absence of *ARP2* editing (*ARP2/ARP2*) or editing of only one *ARP2* allele (*ARP2/arp2*). Together, these results explain the molecular function of ARP2 on the cell periphery and support a functional role for both ARP2 and APR3 components of the ARP2/3 complex in maintaining PEN1 levels in Arabidopsis.

### Identification of *arp2* diallelic heterozygous mutations

**FIGURE 3:**
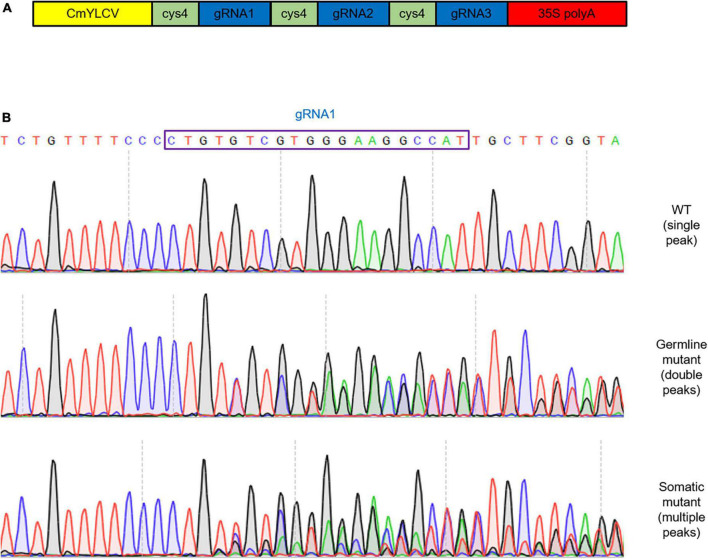
Germline and somatic mutations detected in T1 transgenic plants. **(A)** Structure of multi-array gRNA cassette carrying gRNA1, gRNA2, and gRNA3 to target *ARP2*, and introduced to GFP-PIN1 expressing plants. The CmYLCV promoter and 35S poly A terminator were used. **(B)** DNA sequences of variant deletion from Sanger sequencing of PCR products. The sequences and chromatograms of the unmodified locus for gRNA1 in WT (top), germline mutant line (middle), and somatic mutant line (bottom). gRNA1 target site is marked in a blue box.

**FIGURE 4:**
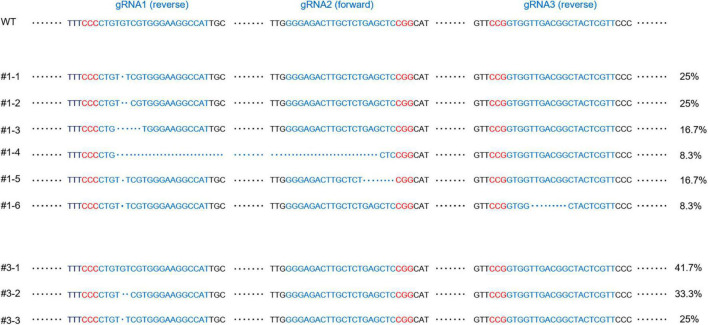
Short and long deletion sequence variants detected in T1 transgenic plants. The sequences of the unmodified locus for gRNA1, gRNA2, and gRNA3 in WT are labeled along the top. gRNA target sites are in blue and PAM sequences are in red. The deletions from T1 transgenic plants #1 and #3 are listed below WT, with generated deletions to gDNA represented by blue dots. #1-1 to #1-6 and #3-1 to #3-3 represent the different PCR products from plant #1 and #3, respectively. The appearance ratios of corresponding sequences in the screening of PCR products are indicated on the right. #3-1 shows no edits on all three gRNA target sites. No fragments like WT or #3-1 were detected in transgenic line #1.

To investigate whether targeted edits were present in these primary T1 CRISPR/Cas9 transgenic plants displaying the trichome defect phenotypes, DNA was extracted for Sanger sequencing from the same first true leaves examined in Figures 1, 2. The extracted DNA was used as templates for the amplification of fragments covering all three gRNA target sites. Through Sanger sequencing of amplified fragments, double or multiple peaks starting from the first gRNA loci (Figure 3) were identified to suggest the production of CRISPR/Cas9-induced gene editing in these plants. Since multiple peaks (more than two) were identified in some plants, we envisioned that besides the germline gene editing (double peaks), divergent somatic edited events (multiple peaks, at least 3 in 36, 8.3% T1 transgenic lines) might also exist in different cells from the first true leaves of these examined plants, resulting in *ARP2* diallelic heterozygous mutations (in which both *ARP2* alleles were differently edited at non-homologous sites of the genic sequences) and displaying the completely or partially aberrant trichomes shown in Figure 1 (plants #1, #2, and #3). To clarify the outcome of gene editing on each positive transgenic plant, the corresponding amplified genomic fragments were cloned, inserted into T-vectors, and sequenced. In this approach, a bulk of plasmids containing different sub-fragments from multiple peaks for each plant were generated and used for Sanger sequencing to assess and confirm the gene editing events. The detailed sequence information covering the three gRNA target sites for two representative somatic edited T1 plants (#1 and #3) is listed in Figure 4. Multiple gene editing events corresponding to gRNA target sites were identified in both #1 and #3 plants, where the editing events on the first gRNA target site (gRNA1) of the gRNA multiplexing cassette show the highest frequency of deletions (100% in #1 and 58.3% in#3), followed by gRNA2 (25% in #1) and gRNA3 (8.3% in #1). These observed edited sequences suggest that the position of gRNA on the multiplexing cassette does have an effect on gRNA activity during the editing process. For all of the 12 sequenced plasmids obtained from plant #1, each of them contains at least one deletion on the gRNA target sites, and no wild-type-like fragments were identified, indicating the complete loss of function of both *ARP2* wild-type alleles in plant #1 and high Cas9 activity in this line. For plant #3, 5 of the 12 sequenced plasmids contained a non-deleted status on the three gRNA target sites compared to the wild-type template. For the other seven sequenced plasmids from plant #3, two different deletions on the gRNA1 target site were detected, indicating the partial gene editing of ARP2 in plant #3. All identified deletions resulted in either frameshift mutations or the loss of amino acid residues (Figure 4). In plant #3, non-deleted fragments were detected in 41.7% of sequenced plasmids, suggesting the existence of homozygous *ARP2*/*ARP2* or heterozygous *ARP2*/*arp2* genotypes in many cells from the first true leaf of line #3. For both #1 and #3 plants, more than one type (six or two) of deletion was detected (Figure 4), indicating that the CRISPR cassette used in this study generated a high ratio of somatic mutations in the cells from the first true leaves of these lines. These somatic mutations are less heritable but are useful to form diallelic heterozygous *arp2*/*arp2* mutations containing cells, which are suitable for early detection of positive targeted edited events and for performing further phenotypic and GFP-PEN1 marker-based analysis. As abnormal trichome is a recessive trait caused by *arp2*/*arp2* allele, cells in the first true leaves of line #3 containing *ARP2*/*ARP2* or *ARP2*/*arp2* alleles can still maintain the normal trichome development and a similar level of GFP-PEN1 signals as wild-type, explaining the partially aberrant trichome phenotypes and relatively stronger GFP-PEN1 signals as shown in Figures 1, 2.

### Effects of actin-related protein 2 disruption on the accumulation of PEN1 at the papillae

**FIGURE 5:**
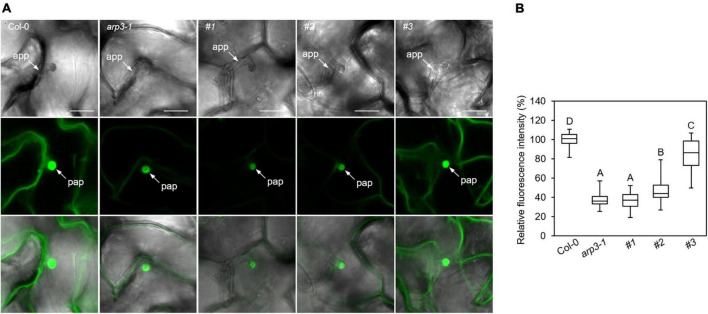
Clustered regularly interspaced short palindromic repeats (CRISPR)-derived variants of *ARP2* exhibit different levels of GFP-PEN1 accumulation at penetration sites upon powdery mildew fungus *Bgh* attack. **(A)** Deposition of GFP-PEN1 at the *Bgh*-attempted penetration sites. Leaves of 4 weeks old from wild-type *Col-0*, *arp3-1* T-DNA Salk line, and T1 generation of CRISPR transgenic individuals #1, #2, and #3 in the GFP-PEN1 expressing background were inoculated with *Bgh* and examined for GFP-PEN1 signals by confocal microscopy at 24 hpi. Bars, 20 μm. Micrographs were captured with the same imaging parameters. **(B)** Boxplots display relative fluorescence intensity of GFP-PEN1 at the papillae in the indicated genotypes. Quantitative analysis was performed for 30 cells per genotype. Different letters indicate statistically significant differences, as determined by one-way ANOVA with Tukey’s HSD (*p* < 0.01). app, appressorium; pap, papilla.

In Arabidopsis, PEN1 mediates a default secretory pathway involved in penetration resistance that involves the focal accumulation of PEN1 at penetration sites (Collins et al., 2003; Assaad et al., 2004). Our results reveal that loss of function of ARP2 in the leaf leads to the aberrant trichome phenotype and less deposition of PEN1 at PM. The focal accumulation of GFP-PEN1 at penetration sites is unaffected by the actin polymerization inhibitor cytochalasin E but requires the actin motor proteins myosins XI and ARP3 (Yang et al., 2014; Qin et al., 2021). To evaluate whether the decreased level of GFP-PEN1 at the PM caused by CRISPR-edited *arp2*/*arp2* mutation contributes to the reduced GFP-PEN1 deposition into papillae upon fungal invasion, we applied the barley–powdery mildew *Bgh* to investigate this question. The true leaves of wild-type, *arp3-1* T-DNA lines, and the CRISPR transgenic lines under GFP-PEN1-expressing background contained GFP-PEN1 signals in the epidermal cells, close to *Bgh*-penetration sites at 24 h after inoculation (hpi) (Figure 5). In wild-type leaves, enhanced GFP-PEN1 signals focally accumulated in the papillae underneath the *Bgh* penetration sites. Conversely, the accumulation of GFP-PEN1 in the papillae was significantly reduced in the *ARP2* CRISPR-edited transgenic mutant lines generated in this study (Figure 5). Line #1 had the greatest decrease in GFP-PEN1 signals in papillae, with signal intensities that were comparable to the GFP-PEN1 signals observed in *arp3-1* T-DNA lines. These data suggest an indispensable role for ARP2 of the ARP2/3 complex in recruiting PEN1 proteins into the apoplastic papillae.

### Validation of the potential off-target sites

In the context of nuclease-based gene drives for target-specific editing through the CRISPR/Cas9 system, genome-editing events can occur at off-target sites, which can produce undesirable genomic engineering outcomes. To reduce off-target editing, we carefully assessed the design of our CRISPR construct to identify potential genomic off-targets and reduce the cleavage at off-target sites with this construct. For the three gRNAs selected for *ARP2* in this study, when specificity was considered, none of them showed any potential genomic off-targets with less than four mismatches (gRNA1 and gRNA2 have no potential off-targets with less than six mismatches). Thus, the potential off-target recognition rates have been significantly decreased by the gRNA design, and in most cases, mutations arising from these events will remain rare and would likely be of no consequence for this study. To investigate whether the only potential off-target gene (AT5G08370), which has 4 bp mismatch with gRNA3 could serve as a target for the CRISPR construct used in this study, we cloned the fragments containing this potential off-target region from six successful T1 *ARP2*-edited lines (including #1, #2, #3) and Sanger sequencing results showed that no gene-editing events occurred in gDNA sequences of AT5G08370. This result supports the absence of CRISPR-induced off-target mutations generated by our construct and plant transformation, affirming the specificity and feasibility of our experimental design to examine the phenotypes through diallelic heterozygous mutations generated by the high-efficiency multiplexing CRISPR/Cas9 system.

### Analysis of stable T2 generation clustered regularly interspaced short palindromic repeats-edited *arp2* mutant lines

**FIGURE 6:**
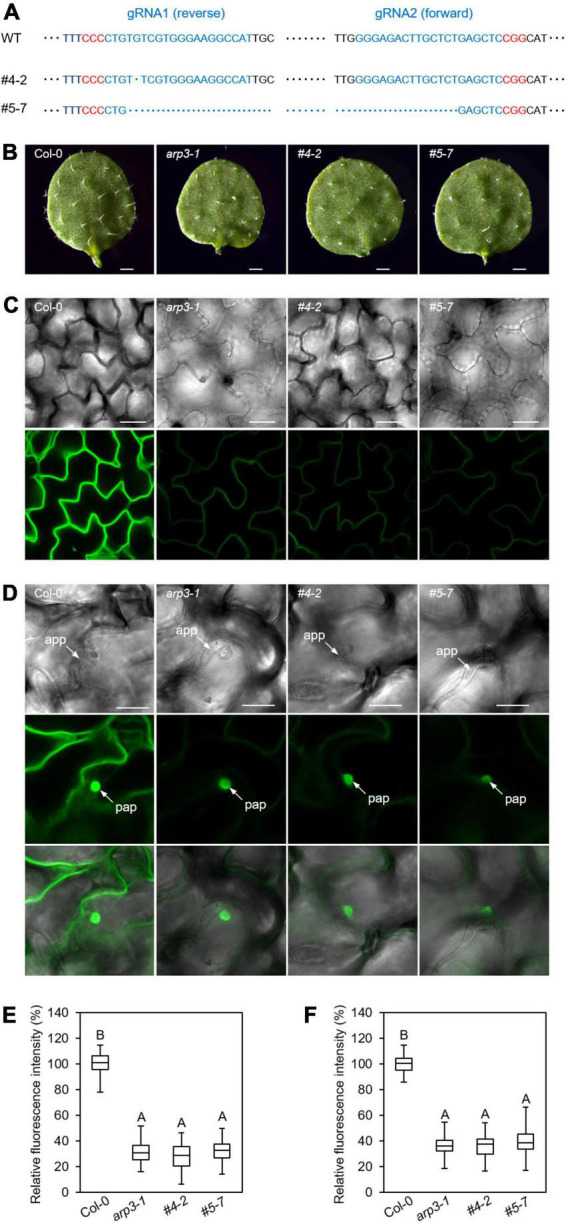
Trichome phenotypes and GFP-PEN1 signals in CRISPR-edited stable T2 transgenic lines. **(A)** Sequence variants of gRNA loci detected in two T2 homozygous transgenic plants. The sequences of the unmodified locus for gRNA1 and gRNA2 in WT are shown on the top. gRNA target sites are in blue and PAM sequences are in red. The deletions in T2 transgenic plants, #4-2 and #5-7, are indicated below the WT by blue dots. The gRNA3 loci from these two plants are both non-edited. In total, 15 of the progenies from these two lines (T3) were sequenced, respectively, and the deletions of gRNA1 and gRNA2 loci are the same as the sequences shown in this figure. **(B)** Overview of trichomes on the first true leaf of 2-week-old plants from WT, *arp3-1* mutant, #4-2, and #5-7 T2 transgenic lines. Bars, 1 mm. **(C)** Representative confocal images of GFP-PEN1 in the leaf epidermis of 2-week-old plants. The images were captured by confocal microscopy with the same setting. Bars, 100 μm. **(D)** Accumulation of GFP-PEN1 at the *Bgh*-attempted penetration sites. Leaves of indicated genotypes were inoculated with *Bgh* and examined for GFP-PEN1 signals by confocal microscopy with the same setting at 24 hpi. Bars, 20 μm. **(E,F)** Boxplots displaying relative fluorescence intensity of GFP-PEN1 signal intensity at the PM **(C)** or papillae **(D)** in the indicated genotypes as described. Quantitative analysis was performed from 30 cells of five plants per genotype. Different letters indicate statistically significant differences determined by one-way ANOVA with Tukey’s HSD (*p* < 0.01).

To further validate the observations from *arp2* somatic mutations in primary T1 generation plants, we have analyzed more transgenic lines produced with ARP2-targeted CRISPR constructs. Among the 36 positive T1 CRISPR transgenic plants produced in this study, two lines were identified as heterozygous germline mutants (*ARP2*/*arp2*, #4 and #5) through Sanger sequencing of PCR products containing three gRNA loci. The T2 progenies of these two transgenic lines showed segregation of ARP2 edited locus, producing two independent homozygous *arp2*/*arp2* mutant lines (#4-2 and #5-7), which were further confirmed by sequencing (Figure 6A). The analysis of results from these progenies confirmed that line #4-2 and #5-7 are the stable *arp2/arp2* CRISPR-edited independent transgenic lines. Therefore, these two lines were used to perform the phenotyping assays for trichome morphology and GFP-PEN1-specific expression patterns as previously performed on somatic mutants identified in T1 plants to validate the molecular function of ARP2. Comparable and severe abnormal trichome phenotypes were observed in these two homozygous germline mutant lines resembling the #1 somatic mutant line in the T1 generation (Figures 6B–F). The T2 stable *arp2*/*arp2* germline mutant lines (#4-2 and #5-7) also exhibited significantly decreased GFP-PEN1 signals at the plasma membrane as well as at the *Bgh*-attempted penetration sites compared to wild-type. These results clearly illustrated the essential molecular and biological functions of ARP2 in Arabidopsis. The T3 generation progenies of #4-2 and #5-7 lines were produced and further sequence verified at three gRNA loci, and all T3 plants carried the same CRISPR-generated mutations as their #4-2 and #5-7 T2 parents, thus confirming the stable inheritance of *arp2*-edited events into the T3 generation. By performing additional molecular analysis, we detected the vector sequence residues at the stable T2 and T3 generations of #4-2 and #5-7 CRISPR-edited mutants. Through PCR analysis using the kanamycin and Cas9-specific primers, we found that the donor vector containing the CRISPR/Cas9 cassette sequences cannot be detected in #4-2 line or its progenies. However, in #5-7 line, the donor vector containing the CRISPR/Cas9 cassette is present as a heterozygous state, and in #5-7’s progenies, we identified segregating *ARP2*-edited mutants without CRISPR/Cas9 sequences in this genetic background. These #4-2 and #5-7 *ARP2* CRISPR-edited lines, in which Cas9/gRNA sequences are deleted, are suitable for the functional analysis of ARP2 because Cas9 nuclease activity is not present anymore in the background to create additional edited events.

### Expression of *ARP2* and *ARP3* and intracellular trafficking of GFP-PEN1 proteins in stable *arp2* clustered regularly interspaced short palindromic repeats mutant lines

**FIGURE 7:**
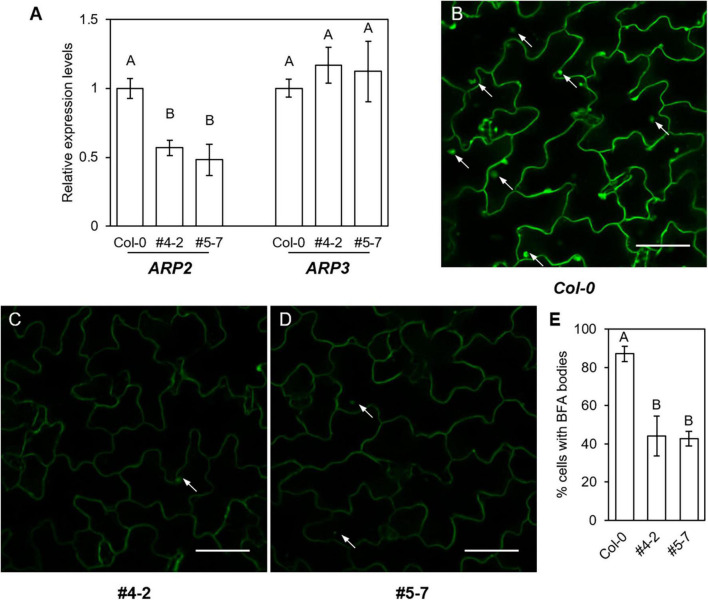
Intracellular trafficking of GFP-PEN1 proteins in CRISPR-edited stable transgenic lines. **(A)** Relative gene expression levels of *ARP2* and *ARP3* at indicated lines expressing GFP-PEN1. Relative expressions were calculated based on the expression of Col-0 expressing GFP-PEN1 lines, the expression of *Actin2* is used as the internal control. Four biological replicates were used for each genotype. **(B–D)** Intracellular aggregation of GFP-PEN1 after BFA treatment in GFP-PEN1 expressing Col-0 **(B)**, ARP2 CRISPR-edited stable lines #4-2 **(C)** and #5-7 **(D)**. BFA bodies were imaged after 60 min 50 μM BFA treatment and indicated by arrows. Bars, 40 μm. **(E)** Percentage of cells showing intracellular accumulation of BFA bodies in indicated lines. At least 300 cells were applied for each genotype. Different letters in panels **(A,E)** indicate statistically significant differences determined by one-way ANOVA with Tukey’s HSD (*p* < 0.01).

To assess whether the CRISPR-edited *arp2/arp2* mutants affect the expression of mutant *ARP2* transcripts and/or *ARP3*, we designed *ARP2*- and *ARP3*-specific primers, and performed targeted-gene expression analysis in the stable *ARP2*-edited independent mutants of #4-2 and #5-7 lines employing Droplet Digital PCR (ddPCR) Assay. In both *arp2/arp2* gene-edited lines, the expressions of *arp2* mutant transcripts are significantly lower compared to their WT transcript levels, while the expressions of *ARP3* remain at similar levels as in the WT (Figure 7A). This result reveals that the loss of function of ARP2 does not alter the expression of ARP3, the other subunit of multimeric ARP2/3 complex, or at least not at the transcriptional level.

The fungal toxin brefeldin A (BFA) has been reported to trigger the accumulation of GFP-PEN1 proteins in intracellular BFA bodies (Nielsen et al., 2012). To determine the potential involvement of ARP2 in maintaining the steady-state level of PEN1 proteins *via* cellular trafficking in Arabidopsis, we examined the cellular distribution of GFP-PEN1 proteins upon BFA-induced GFP-PEN1 accumulation through BFA treatment in WT and both stable *ARP2*-edited mutants of #4-2 and #5-7 lines. After 60 min of BFA treatment, GFP-PEN1 signals aggregate and form large intracellular BFA bodies in most leaf epidermal cells of wild-type plants (87.1%, Figures 7B,E). In contrast, only 44.2% (#4-2) and 42.7% (#5-7) of *arp2/arp2* mutant cells contain BFA bodies (Figures 7C–E). These results illustrate that ARP2 also plays an important role in regulating the intracellular trafficking of GFP-PEN1 to maintain the steady-state level of PEN1 at the PM, implementing a similar functionality as for APR3.

Together, in this study, we have shown the feasibility and usefulness of the CRISPR/Cas9 gene-editing system to produce diallelic somatic mutations in primary T1 generation transgenics, which speeds up the early functional evaluation of edited events for the targeted *ARP2* gene, a component of the ARP2/3 complex. Detailed follow-up confirmatory studies in T2 and T3 generations of stable CRISPR edited *arp2/arp2* plants explain the supporting roles of *ARP2* in regulating PEN1 cellular trafficking and modulating penetration resistance in Arabidopsis against fungal invasion.

## Discussion

Major efforts have been made over the past decades to identify the roles of the actin cytoskeleton during the plant immune responses to pathogen attacks. Recent studies have shown that the formation of actin patches is regulated by the ARP2/3 complex and its activator W/SRC, and *ARP3* is crucial for maintaining the steady-state levels of the defense-associated protein PEN1 at the plasma membrane (Li and Staiger, 2018; Qin et al., 2021). Yet, the role of *ARP2* during this biological process has remained unknown. Addressing this poses a major challenge because of the failure to introduce the GFP-PEN1 marker into *arp2* T-DNA mutant background by genetic crosses. In this study, as an alternate strategy, we have used a high-efficiency CRISPR/Cas9 method (Čermák et al., 2017) to produce different diallelic somatic mutations in the T1 generation and stable lines in the T2 generation, allowing *ARP2* mutation to be combined with the GFP-PEN1-expressing background. With the successful production of the completely or partially mutated *ARP2* lines, we can gain new insights into the functions of ARP2 in Arabidopsis, especially its functions in trichome development and subcellular localization of GFP-PEN1 upon fungal invasion.

### Actin-related protein 2 has similar roles as actin-related protein 3 in Arabidopsis

Maintaining the steady-state level of PEN1 at the plasma membrane and its retargeting to the penetration sites are regulated by continuous endocytosis and GNOM-mediated recycling of pre-synthesized proteins (Nielsen et al., 2012). Our previous work showed that ARP2/3 complex-mediated actin assembly plays a crucial role in modulating this endocytic pathway, and *arp3-1* mutant exhibited reduced GFP-PEN1 signals at the plasma membrane and impaired deposition at the papillae (Qin et al., 2021). In this study, we show that the loss of function of *ARP2* affects the GFP-PEN1 signals at the plasma membrane and papillae, which varied in the degree of severity in different transgenic lines. In the case of plant #1, in which the *ARP2* gene was completely edited with mutations in both alleles, a similar defect in reduced GFP-PEN1 signals was identified as observed in the null *arp3-1* T-DNA mutant (Figures 2, 5).

It has been previously reported that the key subunits of the ARP2/3 complex, ARP2 and ARP3, show some differences in several cellular processes in yeast, although they are indispensable in the initiation of branched filaments. For example, only ARP3 but not ARP2 is necessary for the assembly of the ARP2/3 complex, and *arp3* mutants show more severe defects while *arp2* mutants display moderate defects during the endocytosis in fission yeast (Nolen and Pollard, 2008). However, in Arabidopsis, the present study identified equal importance and essential non-redundant roles for both ARP2 and ARP3. For example, null *arp2* or *arp3* single mutants present the same severity of developmental defects in an array of tip-growing cells, such as trichomes, pavement cells, hypocotyl cells, and root hairs (Mathur, 2005; Szymanski, 2005). Furthermore, both ARP2 and ARP3 are required for the formation of actin patches underneath fungal penetration sites upon powdery mildew attack (Qin et al., 2021). In this study, we demonstrate that ARP2 plays a non-redundant and similar role to ARP3 in the endocytosis of GFP-PEN1 in epidermal cells and in the accumulation of GFP-PEN1 at papillae upon fungal attack. Thus, in contrast to yeast, these findings suggest that both ARP2 and ARP3 are required for the assembly of functional ARP2/3 complex-mediated polymerization of branched actin filaments in Arabidopsis, and thereby raises the possibility for additional plant-specific essential roles for these proteins.

### A clustered regularly interspaced short palindromic repeats design for high-efficiency gene editing

In recent years, advancements in genome engineering with CRISPR-based technologies generated major interest for applications in both fundamental and applied areas of biological research. One of the unique applications is that with CRISPR/Cas9, it is possible to generate targeted knockout mutations in genes, especially when null T-DNA mutants are not available or when genetic linkage prevents the generation of double mutants. Furthermore, CRISPR genome editing can be performed in germline cells (sperms, eggs, or embryos) to induce heritable genetic changes or in somatic cells (other cells) to induce non-heritable transient changes for assessing functions in somatic cells and tissues. To increase the editing efficiency of the *ARP2* target gene in this study, multiplexing and Csy4 spacer-based tools were applied simultaneously in our CRISPR cassette, generating not only a high ratio of *ARP2*-edited plants but also more events for somatic mutations in the same plant. In this case, high-frequency somatic mutations occurring in both alleles would result in diallelic homozygous or heterozygous (mostly heterozygous) mutations, offering a possibility to observe any altered phenotypes in the somatic *ARP2*-mutated cells. These somatic mutations are chimeric/mosaic and often generated when Cas9 is expressed in all somatic cells (as in plant #1) or in some somatic cells (as in plants #2 and #3; Figures 3, 4). Compared to germline mutation, somatic cell editing has received far less attention for potential applications in plant research. In the present study, we specifically consider the impact of somatic genome editing on the future use of CRISPR in host–pathogen interaction studies using a high-efficiency modified CRISPR gene editing vector, which generated at least 8.3% of detectable somatic mutations in the T1 generation of transgenic plants.

Besides addressing the issue of generating GEP-PEN1-expressing lines in *arp2* T-DNA mutant background, our results show the feasibility of performing phenotypic and functional analysis of CRISPR-edited events in the very early first T1 generation of transgenic plants. This approach offers a significant advantage in comparison with the examination of the germline mutation or traditional crossing with T-DNA mutants, which requires at least two generations of growth, and additional screening to obtain homozygous mutants. For certain experiments, for example, live imaging screening for the cellular stress response, heritable mutants are not required, making our time- and cost-efficient method for generating double mutants an attractive alternative that significantly accelerates the experimental process. As host–pathogen interactions involve distinct cells and tissues, the ability to produce diallelic somatic mutations in these cell and tissue types will offer a unique opportunity to perform functional studies. This study shows that CRISPR-edited *arp2* somatic mutations can produce trichome developmental defects and significant reduction of GFP-PEN marker expression in epidermal cells, thus experimentally showing potential applications of this gene-editing approach.

### Opportunities for the clustered regularly interspaced short palindromic repeats-based somatic mutant approach to uncover gene activities with single-cell resolution

Somatic mutations in different living cells provide an opportunity to investigate the impact of editing on a variety of cell types in the same organism. Single-cell CRISPR screens have facilitated the specific CRISPR-driven gene edits and the resulting perturbed gene expression profiles to be assessed, and these approaches have been widely applied in animals and oncology research to conduct comprehensive analyses of cellular phenotypes (Replogle et al., 2020). In plants, this advanced technology has not been exploited yet, but the emerging spatial capture technology offers potential for the applications of this research, especially in relation to plant host–pathogen interaction studies. Whole leaf fixation alongside the use of spatially barcoded mRNA-binding oligonucleotides can be used to spatially capture gene expression information throughout the organ (Giacomello et al., 2017). In this context, our present work demonstrates the generation of diverse somatic mutations of the target gene (*ARP2*) among all or some epidermal cells. In association with emerging spatial capture technology, the use of single-cell CRISPR analyses based on different types of somatic mutation promises to reveal gene product activities and support continued dissection of plant defense responses to biotic stress with high cellular resolution.

## Conclusion

In summary, using a multiplexing gRNA CRISPR gene-editing approach, we have successfully generated multiple unique somatic diallelic heterozygous *arp2/arp2* mutations and stable homozygous germline mutations in Arabidopsis GFP-PEN1-expressing lines. Both the T1 transgenic plants containing somatic *arp2* mutations and T2 stable *arp2* homozygous mutants display overlapping but differentiated trichome morphology defects, and exhibit altered GFP-PEN1 signals at the plasma membrane and papillae. The similarities of these phenotypes between *arp2/arp2* CRISPR mutations and *arp3/arp3* T-DNA mutations suggest shared non-redundant functions of *ARP2* and *ARP3* in Arabidopsis. Overall, our study not only provides a rapid and efficient gene editing method to generate gene knockout mutations, including generating multiple mutated cell types within the leaf, but also provides additional insights into the role of ARP2 in coordinating a subcellular response to fungal invasion in the epidermis.

## Materials and methods

### Plants materials, clustered regularly interspaced short palindromic repeats construction, and transformation

*Arabidopsis thaliana* plants were grown in growth chambers at 16 h light, 22°C and 8 h dark, 20°C; light intensity: 100–120 μmol m^–2^ s^–1^ provided by Philips F32T8/L941 Deluxe Cool White bulbs for the whole life cycle. SALK line mutant *arp2-1* (SALK_003448) and *arp3-1* (SALK_010045) were obtained from the ABRC stock center in the *Col-0* background. The homozygosity of *arp2-1* and *arp3-1* mutants was confirmed by PCR using gene-specific and T-DNA border primers. Arabidopsis transgenic lines tagged with *PEN1* fused fluorescent protein were generated using the pPEN1:GFP-PEN1 construct (Collins et al., 2003).

To create a construct for gene editing of the *ARP2* gene by CRISPR technology, a previous toolkit was applied (Čermák et al., 2017). The pDIRECT22C backbone was used with the Cys4-P2A-AtCas9 module driven by 35S promoter and the cassette of gRNA arrays with Csy4 spacers driven by the CmYLCV promoter for generating loss-of-function mutation of *ARP2* gene with high efficiency. Three gRNAs were selected based on the specificity and applied to the target *ARP2* genomic sequence, and the integration of gRNA arrays to a modified pDIRECT22C vector was performed with a Golden Gate reaction. The constructed vector was introduced into *Agrobacterium tumefaciens* strain *EHA105* and transformed into Col-0 wild-type and plants expressing GFP-PEN1 using floral dipping. The transformants with Col-0 wild-type background were used for trichome morphology analysis and the transformants with GFP-PEN1-expressing background were used for both trichome morphology and all other GFP signal-related analyses. T1 seeds of transgenic plants were selected on Murashige and Skoog medium with kanamycin, and positive transformants were selected for further morphology evaluation and gene editing fragment validation through Sanger sequencing. Among 36 positive T1 plants under GFP-PEN1-expressing background, three lines (8.3%) with somatic mutations and two lines (5.6%) with heterozygous germline mutations (*ARP2*/*arp2*) were identified. The T2 progenies of the two heterozygous germline mutations were further sequenced to screen homozygous germline mutants (*arp2*/*arp2*), and T3 plants were used to perform progeny segregation analysis to validate whether the T2 homozygous germline mutants are stable. *arp3-1* expressing GFP-PEN1 plants were generated by crossing between *arp3-1* T-DNA Salk line and GFP-PEN1-expressing plant under Col-0 background (Qin et al., 2021), and the homozygous plants identified in the F2 generation were used together with above *ARP2* CRISPR transgenic plants for GFP-PEN1 signal comparison. The primers used for CRISPR construction and fragment validation are listed in Supplementary Table 1.

### Light microscopy and imaging analysis

For wild-type and transgenic plants, the first true leaf from 2-week-old plants and the stem from 4-week-old plants were excised and live images were taken with a fluorescence stereo Leica M205 FCA microscope. High magnification images of the trichomes on the leaves and stems were taken and the morphology of trichomes was recorded.

The fluorescence of GFP-PEN1 was detected with the excitation/emission wavelength setting: 488/505–530 nm under confocal laser scanning microscopy (ZEISS LSM 880) using inoculated and non-inoculated leaves, respectively. Images were processed and analyzed with ZEN 3.0 (black version) and Fiji ImageJ software. Quantification of the relative fluorescence intensity of the GFP-PEN1 signals was performed according to the method described previously (Qin et al., 2021). Briefly, the original images were collected with the same acquisition parameters, including laser power, pinhole, detector gains, speed, zoom factor, and resolution. Fluorescence intensity was measured with background subtracted in Fiji ImageJ software. The quantification was conducted for 30 cells per genotype. Statistics analysis was performed using one-way ANOVA with Tukey’s HSD.

### Pathogen inoculation and *in situ* localization of GPF-PEN1 expression

The barley–powdery mildew *Blumeria graminis f. sp. hordei* (*Bgh*) was maintained on barley (*Hordeum vulgare L*, cultivar CDC silky). Arabidopsis plants (4 weeks old) were inoculated with conidiospores at a density of 5–10 conidia mm^–2^. At the indicated time points post-inoculation (24 hpi), the GFP-PEN1 signal was observed by confocal microscopy and recorded with the same parameter setting. The quantification was conducted for 30 cells per genotype. Statistical analysis was performed using one-way ANOVA with Tukey’s HSD.

### Validation of clustered regularly interspaced short palindromic repeats gene-editing

To detect targeted mutagenesis of *ARP2* in CRISPR transgenic lines in *Col-0* and GFP-PEN1 backgrounds, a pair of primers (DG587, DG588) was designed to target *ARP2* genomic DNA sequence covering all three gRNA target sites (Supplementary Table 1). PCR was conducted in 36 positive T1 plants from both transgenic events under *Col-0* and *Col-0* expressing GFP-PEN1 backgrounds, respectively. PCR products (approximately 1.5 kb) were purified and Sanger sequenced, and the lines with heterozygous peaks (two peaks or more peaks) at the gRNA target sites were considered to contain successful editing. These lines were used for further morphology analyses. After examining the trichome morphology and GFP signals on the first true leaf, the same leaves (complete or partially abnormal trichomes) were used for DNA isolation and PCR amplification with DG587 and DG588. The amplified fragments were purified and ligated into the pGEM-T vector. After the transformation of ligation products with *Escherichia coli* competent cells (One shot TOP10), 12 colonies were selected for Sanger sequencing using M13F and M13R primers. Sequencing results were compared with the *ARP2* gDNA sequence to confirm the fraction of different editing events in each collected leaf.

### Off-target prediction and validation

Cas-OFFinder was used to identify potential off-targets^1^ of three gRNA, respectively. As only gRNA3 has a potential off-target with 4 bp mismatch (AT5G08370), a pair of primers (DG614, DG615) targeting this off-target region was designed and used for validation. PCR by this primer pair was performed on six successful *ARP2*-editing lines and amplified fragments were sent for Sanger sequencing to detect gene editing events in AT5G08370 gDNA.

### Droplet digital PCR assay

For performing the quantitative ddPCR-based gene expression assay, leaf total RNA was extracted following the protocol of RNAqueous-micro kit (Ambion, Austin, TX, United States, catalog no. 1927). Extracted RNA was further treated with DNase I (Thermo Fisher Scientific, San Jose, CA, USA) and then reverse transcribed using the SuperScript IV VILO system (Thermo Fisher Scientific, San Jose, CA, United States) according to the manufacturer’s instructions. Transcript abundance was measured using the Bio-Rad QX200 ddPCR System (Bio-Rad, Richmond, CA, United States). In brief, each 20 μl 1 × ddPCR EvaGreen SuperMix reaction mixture containing cDNA templates, forward and reverse primers with optimized concentration was mixed with 70 μl of Droplet Generation oil through Automated Droplet Generator (Bio-Rad, catalog no. 1864101) to generate PCR droplets. From each droplet mix, 40 μl was transferred to a 96-well PCR plate and sealed using PX1 PCR plate Sealer. PCR thermal cycling was optimized, and the amplification signals were read using the QX200 Droplet Reader and analyzed using QuantaSoft software (Bio-Rad) in two-dimensional mode. The primers used for ddPCR validation are listed in Supplementary Table 1.

## Data availability statement

The original contributions presented in this study are included in the article/Supplementary material, further inquiries can be directed to the corresponding authors.

## Author contributions

PG, LQ, RD, and YW conceived and coordinated the study. PG, HN, and HS designed CRISPR constructs and conducted plant transformation and molecular screening. LQ and HS performed the pathogen inoculation and microscopy analyses. PG and LQ performed the data analysis. PG, LQ, TDQ, DX, LVK, YW, and RD prepared and wrote the manuscript. All authors read and approved the final manuscript.

## Footnotes

1
http://www.rgenome.net/cas-offinder/
